# Case of Intus-susceptio, &c.

**Published:** 1840-01

**Authors:** John Fox

**Affiliations:** Surgeon, Cerne Abbas


*A Case of Intus-susceptio, with separation of Five Inches of Intestine followed by complete recovery.* By JOHN FOX, Esq., Surgeon, Cerne Abbas.

H. Diment, aged 16, ate some nuts on the 8th September, felt unwell on the 9th, and was seen by Mr. Fox on the 10th. He had pain about the umbilicus, and had had no motion for twenty-four hours. It is unnecessary for us to particularise symptoms or treatment, suffice to say that the case assumed all the characters of intus-susceptio, and resisted all the remedies directed against that disease by Mr. Fox. But on the 16th, it occurred to Mr. F. to give a trial to inflation. He immediately procured a bladder, and secured one end of it to the nozzle of a pair of bellows, and the other end to a common enema pipe, and having introduced the pipe its full length into the rectum, the bellows were set in motion by his pupil, and inflation forcibly but slowly persevered in for many minutes, until the poor boy complained of a disposition to "break wind," and said that his "belly was very tight." The convolutions of the intestines could be seen and felt distinctly, the arch of the colon most so; the tube was now withdrawn, and to Mr. F.'s great surprise and gratification, in about twenty minutes he said he felt as if he should soon have a stool; he was therefore lifted and supported upon the bedpan, when he passed off wind in large quantities, which in a few minutes was followed by a very copious and liqid evacuation, containing, however, a few hard lumps. He continued, after this, to pass flatus and stools, and to improve, until the 22d, when he was decidedly worse. Mr. Fox left him, supposing the case hopeless. On his arrival next morning, he was agreeably disappointed. He found that:—

"My patient's bowels had been very copiously moved, that he had afterwards slept for nearly two hours, and that he expressed himself as feeling in every respect more comfortable; at the same time, however, his nurse told me she 'supposed he would not last many days longer, as a large piece of his bowels had come away with one of his stools.' I immediately examined the stool, removed the substance alluded to, and having carefully washed it, I found that the woman's suspicions were correct, and that it was indeed a portion of one of the intestines, with some of the mesentery still adhering."

The lad recovered perfectly. Mr. Fox naturally regrets that he did not resort to inflation earlier.—*Medico-Chirurgical Review.*

